# Comparison of tumor and two types of paratumoral tissues highlighted epigenetic regulation of transcription during field cancerization in non-small cell lung cancer

**DOI:** 10.1186/s12920-022-01192-1

**Published:** 2022-03-21

**Authors:** Qiushi Wang, Libo Wu, Jiaxing Yu, Guanghua Li, Pengfei Zhang, Haozhe Wang, Lin Shao, Jinying Liu, Weixi Shen

**Affiliations:** 1 Department of Thoracic Surgery, The Second Affiliated Hospital of Harbin Medical University, Harbin, China; 2 Burning Rock Biotech, Guangzhou, China; 3 Department of Medical Oncology, The Second Affiliated Hospital of Harbin Medical University, Harbin, 150001 China

**Keywords:** NSCLC, Field cancerization, DNA methylation, Bisulfite sequencing, Epigenetics

## Abstract

**Background:**

Field cancerization is the process in which a population of normal or pre-malignant cells is affected by oncogenic alterations leading to progressive molecular changes that drive malignant transformation. Aberrant DNA methylation has been implicated in early cancer development in non-small cell lung cancer (NSCLC); however, studies on its role in field cancerization (FC) are limited. This study aims to identify FC-specific methylation patterns that could distinguish between pre-malignant lesions and tumor tissues in NSCLC.

**Methods:**

We enrolled 52 patients with resectable NSCLC and collected resected tumor (TUM), tumor-adjacent (ADJ) and tumor-distant normal (DIS) tissue samples, among whom 36 qualified for subsequent analyses. Methylation levels were profiled by bisulfite sequencing using a custom lung-cancer methylation panel.

**Results:**

ADJ and DIS samples demonstrated similar methylation profiles, which were distinct from distinct from that of TUM. Comparison of TUM and DIS profiles led to identification of 1740 tumor-specific differential methylated regions (DMRs), including 1675 hypermethylated and 65 hypomethylated (adjusted P < 0.05). Six of the top 10 tumor-specific hypermethylated regions were associated with cancer development. We then compared the TUM, ADJ, and DIS to further identify the progressively aggravating aberrant methylations during cancer initiation and early development. A total of 332 DMRs were identified, including a predominant proportion of 312 regions showing stepwise increase in methylation levels as the sample drew nearer to the tumor (i.e. DIS < ADJ < TUM) and 20 regions showing a stepwise decrease pattern. Gene set enrichment analysis (GSEA) for KEGG and GO terms consistently suggested enrichment of DMRs located in transcription factor genes, suggesting a central role of epigenetic regulation of transcription factors in FC and tumorigenesis.

**Conclusion:**

We revealed distinct methylation patterns between pre-malignant lesions and malignant tumors, suggesting the essential role of DNA methylation as an early step in pre-malignant field defects. Moreover, our study also identified differentially methylated genes, especially transcription factors, that could potentially be used as markers for lung cancer screening and for mechanistic studies of FC and early cancer development.

**Supplementary Information:**

The online version contains supplementary material available at 10.1186/s12920-022-01192-1.

## Background

Lung cancer is the leading cause of cancer deaths worldwide. Its high mortality is partially attributable to the scarce knowledge of molecular mechanisms mediating lung cancer pathogenesis and the late diagnosis of the majority of lung cancers [1]. Non-small cell lung cancer (NSCLC), representing the majority of diagnosed lung cancers, is a complex malignancy that develops through progressive pathologic changes driven by an interplay of a variety of molecular pathways including both genetic and epigenetic mechanisms [2]. Therefore, mapping genetic and epigenetic changes in normal tissue at high risk of malignant transformation is critically important for understanding the mechanism of carcinogenesis, identifying early causal drivers and predicting cancer risk.

Field cancerization (FC), also referred to as pre-malignant field defect, is the process in which a population of normal or pre-malignant cells is affected by oncogenic alterations leading to progressive molecular changes that drive their malignancy [2, 3]. The acquirement of tumor-primed genetic alterations (such as *EGFR* and *KRAS* mutations, loss of heterogeneity of chromosomal regions 3p and 9p, and genomic instability) has been described in histologically normal bronchial epithelia adjacent to the lung carcinoma [4–6]. On the other hand, DNA methylation is a primary epigenetic modification in the mammalian genome. Aberrant DNA methylation has been implicated in early cancer development, including lung cancer [7]. Belinsky and colleagues reported aberrant promoter methylation of p16, which commonly occurred in lung tumors [8], in bronchial epithelial sites from 44% of lung cancer patients and cancer-free smokers [9]. The aberrant methylation of various frequently methylated genes in lung cancer, including retinoic acid receptor 2 β (*RAR-β2*), H-cadherin, adenomatous polyposis coli (*APC*), and Ras association domain family member 1 (*RASSFF1A*), has also been described in bronchial epithelial cells of heavy smokers [10]. Despite a number of studies [11, 12] have suggested the phenomenon of epigenetic FC in lung cancer, most of them interrogated the methylation profile in pre-malignant lesions (such as basal cell hyperplasia, squamous metaplasia, dysplasia), lacking appropriated subject-matched controls for both normal and malignant tissues. Furthermore, the vast majority of these studies focused on a limited number of candidate genes, the methylation of which had been often observed in lung cancer; the methylation profile was often assessed qualitatively not quantitatively. These limitations would attenuate the measured magnitude of epigenetic differences and inhibit the ability to identify the earliest methylation alterations that occur in carcinogenesis. Given the ubiquitous inter-patient heterogeneity, the extent to which DNA methylation profiles modify the FC effect of individuals may be largely obscured by such study design.

In the present study, we aimed to identify tumor-specific methylation patterns that could distinguish between pre-malignant normal lesions and tumor tissues and could potentially be developed as biomarkers, using subject-matched surgically-resected tumor, tumor-adjacent normal (ADJ) and tumor-distant normal (DIS) tissue samples.

## Methods

### Patients’ information and study design

A total of 52 patients with early-stage resectable NSCLCs from 2018 and 2019 were enrolled in this study. Matched surgically-resected tumor, tumor-adjacent normal (ADJ) and tumor-distant normal (DIS) tissue samples were collected from each patient during surgery. ADJ tissues were biopsied 2 cm distant from resection margins, which allowed for both proximity to the tumor and a low chance of tumor cell contamination in case of a R2 resection margin; DIS samples were biopsied 5 cm distant from resection margins to make sure sample collection was feasible for centrally located stage T4 tumors treatment with surgery of curative intent, which usually sets the margin ~ 5 cm outside of the tumor zone. Samples underwent histopathological assessment. Tumor tissues with a tumor cell fraction < 10% and normal tissues with any visible tumor cell were excluded. Eventually, thirty-six patients had all three types of samples subjected to bisulfite DNA sequencing for methylation profiling that was used for subsequent analyses to identify differential methylation signatures. The histopathological and clinical characteristics of patients were collected. The study was approved by the institutional review board of The Second Affiliated Hospital of Harbin Medical University. All patients provided written informed consent, in accordance with the Declaration of Helsinki.

### DNA isolation

Genomic DNA were extracted tissue samples using a QIAamp DNA FFPE tissue kit, according to the manufacturer’s standard protocol (Qiagen, Hilden, Germany). DNA was quantified using the Qubit dsDNA assay (Life Technologies, Carlsbad, CA, USA).

### Bisulfite targeted sequencing

DNA was sequenced using a brELSATM method as described previously [13]. Briefly, purified DNA was converted to single-strand DNA by sodium bisulfite treatment. The converted single-strand DNA was subsequently ligated to a splinted adapter, and amplified by a uracil-tolerating DNA polymerase to generate whole-genome bisulfite sequencing (BS-seq) libraries. Target enrichment was performed using custom-designed lung-cancer methylation profiling RNA baits covering 80,672 CpG sites, spanning 1.05 megabases of the human genome (Burning Rock Biotech, Guangzhou, China). The target libraries were finally quantified by real-time PCR (Kapa Biosciences, Wilmington, MA, USA) and sequenced on a NovaSeq 6000 (Illumina, San Diego, CA, USA) using 2 × 150 bp cycles. The targeted methylation panel was designed as previously described [14, 15]. Briefly, many differentially methylated loci (DMLs) were selected from the 450 K microarray data of NSCLC/ adjacent tissue and normal plasma samples downloaded from TCGA dataset.

### Data analysis

Bisulfite sequencing data analysis was performed using an optimized pipeline. Trimmomatic (v.0.32) was used to remove custom adaptor sequences and low-quality bases. Paired-end reads were aligned to C to T- and G to A-transformed hg19 genome using BWA-meth (v.0.2.2) [16]. After alignment, duplicate reads were marked by samblaster (v.0.1.20) [17], and low mapping quality (MAPQ < 20) or improper pairing reads were removed by sambamba (v.0.4.7) [18] from downstream analyses. Paired reads were merged by clipping overlapping reads to avoid double-counting of methylation calls.

### Identification of differential methylation regions (DMRs)

**Fig. 1:**
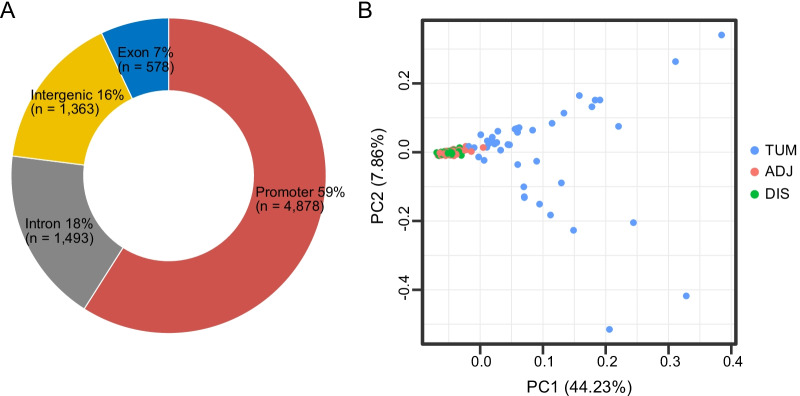
PCA analysis of methylation signatures in tumor tissues (TUM), adjacent (ADJ) and distant histologically-normal tissues (DIS). (**A**) The distribution of 8312 blocks in genome; (**B**) PCA analysis based on the methylation signatures of 8312 blocks

The 80,672 CpG sites included in the panel were grouped into 8312 methylation blocks using an algorithm as described previously [15]. Specifically, we applied a region-defined algorithm with co-methylation effect between adjacent CpG sites in consideration [14]. To estimate the predefined coefficients of the algorithm, we used a series of methylation data of different tissues with the same panel mentioned in this study. Methylation blocks were defined as the genomic region consisting of the neighboring CpG which were not only close on distance but also correlated on methylation level. Briefly, the difference among the methylation frequencies of each pair of CpG sites was calculated by Pearson’s correlation analysis, and normalized by the difference in genomic distance and methylation level. Within 8312 blocks, 84% were annotated in genes with 59% in promoter regions, 7% in exons and 18% in introns (Fig. 1A).

A block-wise statistic methylMean was generated for downstream analyses. Besides the CpG sites, the information of CHH (H denotes A, T or C) sites were also included to estimate the background error in sequencing which would help to correct the methylMean value of CpG sites. We denote *M*_*j*_/*U*_*j*_ as the number of methylated/unmethylated read counts for the *j*th CpG sites within a methylation block. And *Me*_*k*_/*Ue*_*k*_ denotes the number of methylated/unmethylated read counts for the *k*th CHH sites within a methylation block. The corrected methylMean was defined as

$$\begin{aligned} methylMean & = \frac{{\mathop \sum \nolimits_{j} M_{j} }}{{\mathop \sum \nolimits_{j} (M_{j} + U_{j} )}} \\ error & = \frac{{\mathop \sum \nolimits_{j} Me_{j} }}{{\mathop \sum \nolimits_{j} (Me_{j} + Ue_{j} )}} \\ corrected\,methylMean & = \frac{methylMean - error}{{1 - error}} \\ \end{aligned}$$

The differential methylated regions (DMRs) were identified by comparing corrected methylMean values of blocks between different groups using “limma” package in R software. Blocks with significant difference (threshold abs (log2FC) > 0.1, adjust p-value < 0.05) were chosen. Bonferroni correction was applied to for multiple comparisons. The volcano plot and heatmap were drawn using R software.

### Functional enrichment analyses

Gene Set Enrichment Analysis (GSEA) [19, 20] was performed for the functional annotation of DMRs using the Molecular Signatures Database (version 7.4) [21]. For KEGG terms, c2.cp.kegg.v7.4.entrez.gmt and c2.cp.v7.4.entrez.gmt were used separately [22]. Gene Ontology (GO) Enrichment Analysis was also performed for DMRs [23]. The cut-off of two-sided adjusted p value (i.e. false discovery rate) was set to 0.05.

### Statistical analysis

Statistical analysis was performed using R version 3.3.3 software. Principal component analysis (PCA) [24] and hierarchical clustering analysis were performed for clustering samples according to their methylation profiles using all 8312 blocks or tumor specific block. Differential methylation analysis was performed with the “limma” package. Differences were evaluated with Fisher’s exact test for proportions of categorical variables across groups, with Pearson’s correlation analysis for 2 continuous variables, and with paired Student’s t-test for DNA methylation levels between 2 groups and multiple paired t-test for 3 groups. For other continuous variables between 2 groups, the Wilcoxon rank sum test was used for comparison, and ANOVA was performed for continuous variables across 3 groups. Statistical significance was defined as two-sided P values < 0.05.

## Results

### Demographic and clinicopathological characteristics of patients

Three types of tissue samples, including surgically-resected tumor (TUM), tumor-adjacent normal (ADJ) and tumor-distant normal tissue (DIS) were collected from 52 enrolled NSCLC patients. Among them, 36 generated sequencing data with sufficient quality for all 3 sample types samples and therefore underwent further analyses. The demographic and clinicopathological characteristics of the 36 patients were summarized in Table 1. The median age of the cohort was 58.6 years, ranging from 30 to 73. Male and female patients comprised 52.8% and 47.2% of the cohort, respectively. Of the 36 patients, 16 (44.4%) had no smoking history, and 6 (16.7%) and 14 (38.9%) patients were former and current smokers, respectively. Ten patients (27.8%) had their tumors measuring < 5 cm^2^; 12 (33.3%) had tumors measuring 5–9 cm^2^; and the tumor size of 11 patients (30.6%) ranged from 10 to 20 cm^2^. Only 3 patients had tumors > 20 cm^2^. The majority of the patients (50%) had adenocarcinomas, 19.4% were diagnosed with squamous cell carcinomas, and 30.6% with tumors of other histology. The T stage and N stage were also summarized in Table 1.

**Table 1:** Characteristics of the 36 patients with qualified bisulfite sequencing data for matched TUM, ADJ and DIS samples

Characteristic	No. of patients (%)
Age, years (Median [Range])	58.6 [30–73]
Sex	
Male	19 (52.8)
Female	17 (47.2)
Smoking status	
Never	16 (44.4)
Former	6 (16.7)
Current	14 (38.9)
Tumor size (cm^2^)	
< 5	10 (27.8)
5–9	12 (33.3)
10–20	11 (30.6)
> 20	3 (8.3)
Histology	
ADC	18 (50.0)
SCC	7 (19.4)
Others	11 (30.6)
T stage	
T1	12 (33.3)
T2	11 (30.6)
T3	6 (16.7)
T4	6 (16.7)
Unknown	1 (2.8)
N stage	
N0	22 (61.1)
N1	10 (27.8)
N2	3 (8.3)
Unknown	1 (2.8)

The 52 enrolled patients underwent exclusion by tumor cell fraction within their TUM, ADJ, and DIS samples, and 36 were eligible for subsequent analyses. ADJ—tumor-adjacent normal tissue. DIS—tumor-distant normal tissue. TUM—surgically-resected tumor

### Distinct methylation profile of tumor tissues

PCA analysis was first performed based on 8312 blocks and demonstrated the distinct methylation profile of TUM as compared to both ADJ and DIS tissues (Fig. 1B). Meanwhile, ADJ and DIS tissues had a similar methylation profile. Furthermore, heterogeneous methylation profiles were observed within tumor tissues.

**Fig. 2:**
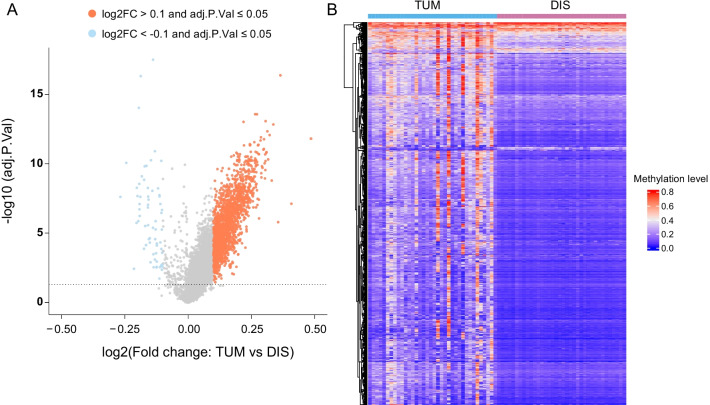
Differentially methylated regions (DMRs) in tumor tissues (TUM) as compared with distant normal tissues (DIS). (**A**) The volcano plot of cancer-specific methylation blocks. (**B**) The heatmap of the 1740 tumor-specific DMRs

A total of 1740 tumor-specific DMRs, including 1675 hypermethylated and 65 hypomethylated DMRs, spanning 626 genes were found to be differentially methylated in TUM as compared to DIS (abs (log2FC) > 0.1, adjusted p-value < 0.05, Fig. 2, Additional file 1: Table S1). Six of the top 10 differentially hypermethylated genes have been associated with lung cancer (Table 2), including *BARHL2 DMRTA2*, *OTX1*, *OTX2*, *MIR124* and *HOXA9*.

**Table 2:** The top 10 hypermethylated genes in tumor tissues

Gene	log2(Fold change)	p value	Adjusted p value
*BARHL2*	0.486	2.42E − 15	1.55E − 12
*MIR124-3*	0.408	4.31E − 09	7.68E − 08
*DMRTA2*	0.337	1.60E − 16	1.48E − 13
*ESPN*	0.330	3.32E − 11	1.63E − 09
*OTX2*	0.322	1.14E − 15	8.60E − 13
*YAE1D1*	0.316	2.83E − 13	4.90E − 11
*SKOR1*	0.313	5.53E − 16	4.60E − 13
*ZNF876P*	0.310	4.89E − 14	1.45E − 11
*HOXA9*	0.305	3.57E − 12	2.85E − 10
*OTX1*	0.305	9.17E − 14	2.12E − 11

**Fig. 3:**
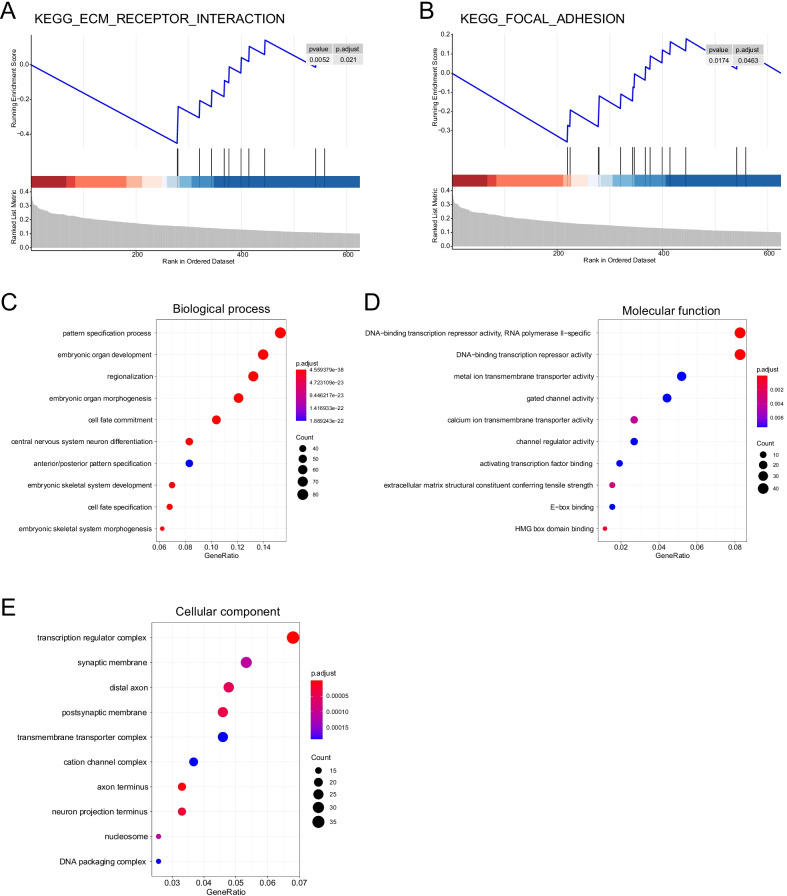
Functional annotation of tumor-specific differential methylation regions. GSEA enrichment analyses identified significantly enriched KEGG pathways **A** extracellular matrix (ECM)-receptor interaction and **B** focal adhesion among genes [22] with lower DNA methylation level in tumor tissues than in normal tissues. The top 10 enriched GO **C** biological process, **D** molecular function, and **E** cellular component terms consistently showed predominance of terms related to transcriptional regulation and chromatin remodeling

Next, we performed both GSEA and GO enrichment analyses for the functional annotation of the DMRs. GSEA analysis demonstrated that genes in the extracellular matrix (ECM)-receptor interaction (NES = −1.89, P = 0.005, adjusted P = 0.021) and focal adhesion (NES = −1.67, P = 0.017, adjusted P = 0.046) pathways were less commonly methylated in tumor tissues than in normal tissues (Fig. 3A, B; Additional file 1: Fig. S1 and Table S2). Besides, GO analysis identified a total of 521 biological processes (BP), 30 cellular components (CC), and 19 molecular functions (MF) enriched among the DMRs. The most significantly enriched BP terms appeared to be related to cell differentiation, including pattern specification process, regionalization, and cell fate commitment (Fig. 3C). The transcriptional machinery was strongly implicated in enriched MF terms (Fig. 3D), which was in line with CC terms enriched in transcriptional regulation complex, transmembrane transportation, and chromatin remodeling (Fig. 3E). Together, these terms depicted a rough picture in which the DMRs participated in an orchestrated program of transcriptional regulation, thereby highlighting the significance of transcription factors and chromatin remodelers in tumor initiation and development. We also performed gene set enrichment separately in genes harboring hyper- and hypomethylated DMRs. Similar enriched terms were observed for the former set (n = 626; Additional file 1: Fig. S2), while no term was significantly enriched among the latter, perhaps due to the small set size (n = 52).

### The identification of field cancerization (FC)-specific DMRs

**Fig. 4:**
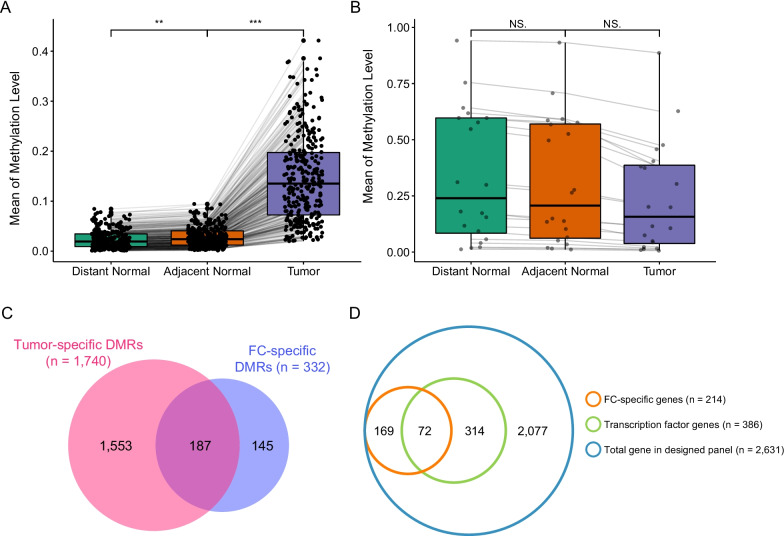
Field cancerization (FC)-specific differentially methylated regions (DMRs). **A** DMRs with methylation level: tumor-distant normal tissues < tumor-adjacent normal tissues < tumor tissues; **B** DMRs with methylation level: tumor-distant normal tissues > tumor-adjacent normal tissues > tumor tissues; **C** The overlap of FC-specific DMRs with tumor-specific DMRs; **D** Enrichment of transcriptional factors in genes differentially methylated in tumor-adjacent normal tissues

In order to further identify aberrant methylation during different steps of cancer development, we compared the methylation level of each block among TUM, ADJ, and DIS by multiple paired-t test. A total of 332 DMRs were found to be differentially methylated among the three tissue types, indicating pre-malignant field-related methylation patterns. The methylation levels from 312 DMRs were significantly lower in DIS as compared to ADJ and also lower in ADJ than in TUM (Fig. 4A). Meanwhile, methylation levels from 20 DMRs were higher in DIS than ADJ, and also higher in ADJ as compared with TUM (Fig. 4B). Among the 332 FC-specific DMRs, 187 (56.3%) were overlapped with tumor-specific DMRs (Fig. 4C). Among the top 15 FC-specific hypermethylated genes, the methylation of *ZSCAN31* [25], *KCNA3* [26] and *CDO1* [27, 28] were reported to be associated with lung cancer development (Table 3). Besides, methylation of *DRD4* [29, 30], *ZNF132* [31] and *ZNF43* [32] have been reported to play roles in other cancer types. Due to the small number of the FC-specific DMRs identified, functional enrichment analyses failed to identify any enriched pathways.

**Table 3:** The top 15 hypermethylated genes in field cancerization

Gene	log2 (Fold change)	P value	adjusted p value
*ZSCAN31*	5.079	< 0.001	< 0.001
*ZNF345*	4.431	0.040	0.022
*DRD4*	4.312	< 0.001	< 0.001
*RAI1*	4.097	< 0.001	< 0.001
*ZNF132*	4.083	< 0.001	< 0.001
*ZNF175*	3.870	0.040	0.023
*ZNF43*	3.861	0.016	0.002
*SNX32*	3.847	< 0.001	< 0.001
*FAM19A2*	3.742	< 0.001	< 0.001
*HIST1H2BE*	3.651	0.040	0.007
*FABP5*	3.541	0.040	0.010
*NTMT1*	3.501	0.040	0.040
*ENPP2*	3.440	0.040	0.032
*KCNA3*	3.401	0.035	0.005
*CDO1*	3.395	< 0.001	< 0.001

The total of 8312 blocks included in the panel span 2631 genes, among which 385 (14.6%) are transcription factor genes. On the other hand, the 332 FC-specific DMRs were annotated in 241 genes. Among the 241 genes, 72 (29.9%) were transcription factor genes (Fig. 4D; Additional file 1: Table S3). Hypergeometric analysis revealed that these differentially methylated genes were enriched with transcription factor genes (P = 4.729e − 11), which were consistent with GO enrichment results. Transcription factors also account for a similar proportion in differentially methylated tumor-specific genes (30%). These remarkable percentages suggested the role of epigenetic regulation of transcription factors as a key step in driving malignancy and FC.

**Fig. 5:**
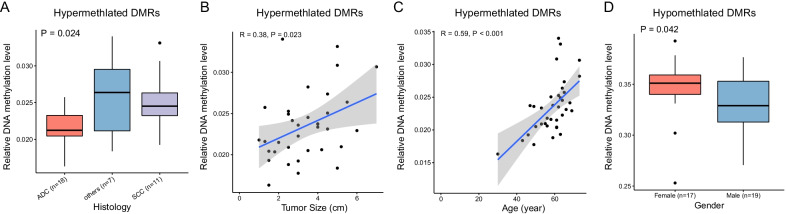
Association between DNA methylation levels of field cancerization-specific differentially methylated regions (DMRs) and clinicohistologic characteristics. (**A**) Relative DNA methylation levels per histology among hypermethylated DMRs. (**B**) Correlation between relative DNA methylation evels and tumor size or (**C**) patient age among hypermethylated DMRs. (**D**) Relative DNA methylation levels per sex among hypomethylated DMRs. Hypermethylated DMRs refer to those showing a methylation level pattern of tumor-distant normal tissues < tumor-adjacent normal tissues < tumor tissues, and hypomethylated DMRs refer to those with a complete reversed pattern. *ADC* adenocarcinoma, *SCC* squamous cell carcinoma

Finally, we evaluated the associations between methylation levels of FC-specific DMRs in tumor samples and clinical characteristics by PCA analysis. We found that age, histology, and tumor size were significantly associated with DMR methylation level (Table 4). These associations were confirmed with further analyses (Fig. 5). Among the genes most intensely methylated in tumor samples, patients with squamous cell carcinoma had significantly higher methylation level than those with adenocarcinoma (P = 0.024; Fig. 5A), and methylation was also significantly correlated with tumor size (R = 0.38, P = 0.023) and age (R = 0.59, P < 0.001; Fig. 5B, C). Among the hypomethylated genes, men showed lower methylation levels than women (P = 0.042; Fig. 5D).

**Table 4:** PCA of the methylation levels of field cancerization-specific DMRs in tumor samples revealed significant associations (in bold) with some clinical features

P value	PC1	PC2	PC3
Age	**0.007**	**1.081e** − **13**	**3.091e** − **05**
Sex	0.374	0.1621	**0.0026**
T-stage	0.1714	0.9696	0.054
N-stage	0.2857	0.2769	0.9209
Histology	0.0728	**0.0003**	0.0792
Smoking status	0.2904	0.7241	0.3701
Tumor size	**0**	0.223	**0**

ADJ—tumor-adjacent normal tissue, DIS—tumor-distant normal tissue, DMR—differential methylation region, TUM—surgically-resected tumor

## Discussion

DNA methylation, occurring very early in the process of carcinogenesis, has been widely recognized as an important cancer-related biomarker. In the present study, we identified 1675 hypermethylated and 65 hypomethylated tumor-specific DMRs, which were annotated by 626 genes. Among these differentially methylated genes, some have been confirmed to be regulated by methylation in lung cancer development. *BARHL2* [33, 34], *DMRTA2* [33, 35, 36], *OTX1* [33, 34] and *OTX2* [33, 37] were identified as DNA methylation markers for lung cancer. Increased methylation of *MIR124* has also been found in NSCLC [38]*.* Methylation of *HOXA9* has been demonstrated as a reliable prognostic marker for NSCLC [28, 39–41]. We further performed functional enrichment analysis to clarify the role of methylation in NSCLC. We found a trend of methylation down-regulation for genes in ECM-receptor interaction pathway (adjusted p = 0.021). ECM constitutes the main part of the extracellular microenvironment. Its synthesis, distribution, and degradation are closely linked to the differentiation, proliferation, invasion, and metastasis of malignant tumors. Overexpression of the ECM-receptor (*hyaluronan receptor HMMR*) has been found primarily in LUAD and was connected with an inflammatory molecular signature and poor prognosis [42]. Lim et al. developed a signature based on the expression of 29 ECM‑associated genes to predict the prognosis of the patients at the early stage of NSCLC [43].

More interestingly, we also identified 332 field-cancerization specific DMRs spanning 241 genes, which were differentially methylated in ADJ as compared with TUM and DIS. Compared with tumor-specific DMRs, these FC-cancerization specific DMRs may represent earlier methylation alterations that occur in carcinogenesis and serve as more sensitive biomarkers for early-detection and risk assessment for lung cancers. Of the 15 genes where the top differentially methylated DMRs residue (Table 3), *ZSCAN31* is thought to function as a transcription factor which is involved in airway structure or remodeling [44]. *ZSCAN31* has also been reported to be significantly hypermethylated in lung cancer [25]. *CDO1* silencing promotes proliferation of NSCLC by limiting the futile metabolism of cysteine. Methylation of *CDO1* has been identified as a specific marker for lung cancer diagnosis [27, 28]. *KCNA3* functions in voltage-gated potassium channels, which play a variety of roles in cancer progression. *KCNA3* inactivation via promoter hypermethylation has been found across multiple cancer types including lung, breast, pancreas, ovarian, kidney, prostate, and colon [26, 45]. Besides, *DRD4*, *ZNF132* and *ZNF43* have been linked with other non-lung cancer types: *DRD4*, encoding dopamine receptor, is involved in early brain development and epigenetically repressed in pediatric CNS tumors [30]; it also has be identified as a potential epidriver in hepatocellular carcinoma [29]. *ZNF132* belongs to C2H2 zinc finger protein family and plays an important role in ESCC development as a tumor suppressor gene. It has been identified as a novel hypermethylation biomarker in ESCC [31]. Hypermethylated *ZNF43* has been reported as a biomarker for colorectal cancer [32]. We reported the first clinical evidence that methylation of *DRD4*, *ZNF132* and *ZNF43*t may be also involved in lung cancer development. Furthermore, we also identified several novel methylation biomarkers that have not previously been reported in cancer. Some of them are transcription factors, such as *ZNF345* and *ZNF175* (Table 3). Hypergeometric analysis further revealed an enrichment of transcription factor genes (p = 4.729e − 11) in the 241 differentially methylated genes. Consistently, the most significantly enriched GO BP, CC, and MF terms highlighted a sizable proportion of genes participating in transcription regulation and chromatin remodeling. Collectively, these findings suggested the role of epigenetic regulation of transcription factors as a key step in driving malignancy and FC.

Different cell type composition could be a powerful source of DNA methylation level changes between surgical samples, and immune infiltration cell is a prominent cause of cell type composition perturbations. To evaluate the extent to which immune infiltration could have affected methylation level changes, we compiled a list of 782 immune cell marker genes from literature. Twelve of these markers harbored regions among the 1675 hyper-methylated regions in this study, and 2 markers for the 65 hypomethylated.

Overall, DM immune cell markers accounted for 0.8% of all DM genes. Additionally, no GO or KEGG terms related to immune-related biological processes or functions were significantly enriched differentially methylated genes (Fig. 3). These findings suggested that immune cell infiltration was present but did not remarkably interfere with the identification of differentially methylated genes. Apart from cell type makeup, functional enrichment of genes identified with a targeted approach may be largely affected by the targeted panel. We compared the enriched GO BP terms among the genes that harbored the targeted methylation blocks and those among randomly sampled subsets (Additional file 1: Fig. S3). The different enrichment results suggested no inherent concentration of specific GO terms among the 2613 targeted genes.

Compared with previous works that identified genes differentially methylated in TUM and ADJ [15, 46, 47], strength of this study partly stems from a novel design that used two normal samples at defined distances way from resection margins and therefore allowed identification of genes that showed FC-specific dynamics of DNA methylation levels. These genes showed progressively enhanced or attenuated methylation as the location drew nearer to the tumor, thereby providing candidate markers for tracking tumorigenesis and early development. On the other hand, the major limitation of this study is the lack of validation of the prognostic values of DMRs. This is largely due to the short follow-up time after the surgery so that the relapse-free survival data of patients remains immature. This study was also limited by the lack of a well-defined consensus on the area undergoing cancerization, as there is clinical evidence suggesting that lung tissues deemed non-tumorous patients NSCLC patients may already be under FC due to carcinogen exposure [46, 47]. Therefore, although the DM genes with progressive increase/decrease as location sample drew nearer to the tumor remain FC-specific, it is unclear how their methylation levels change during early cancer development without a validated non-cancerous control sample. Besides, functional studies should be performed to confirm the roles of those newly identified biomarkers in field cancerization of lung cancer.

## Conclusions

In conclusion, our data revealed distinct methylation patterns between pre-malignant lesions and malignant tumors, suggesting the essential role of DNA methylation as an early step in pre-malignant field defects. Moreover, our study also identified cancer-specific methylation blocks that could potentially be used as markers for lung cancer screening.

## Supplementary Information

**Additional file 1. Fig. S1**: Significantly enriched KEGG pathways terms [1] among tumor-specific differentially methylated genes using a different gene set annotation file showed similar results as in Figures 3A and 3B. All analysis parameters were the same except for the gene set file (c2.cp.v7.4.entrez.gm in this analysis).

**Additional file 2. Fig. S2**: Significantly enriched GO terms among genes harboring hypermethylated field cancerization-specific differentially methylated regions.

**Additional file 3. Fig. S3**: Enriched GO biological process terms among (A) all 2613 genes that harbored the targeted methylation blocks and those among randomly sampled subsets of (B) 100, (C) 200, and (D) 500 genes.

**Additional file 4. Table S1**: Details of tumor-specific differentially methylated regions.

**Additional file 5. Table S2**: Results of GSEA analysis using different MsigDB gene sets. Analysis 1 used “KEGG gene sets as NCBI (Entrez) Gene IDs” (c2.cp.kegg.v7.4.entrez.gmt), and the corresponding significantly enriched KEG pathways are shown in Figures 3A and 3B. Analysis 2 used ll canonical pathways as NCBI (Entrez) Gene IDs (c2.cp.v7.4.entrez.gmt), and the corresponding significantly enriched KEGG pathways aer shown in Figure S1).

**Additional file 6. Table S3**: List of 72 transcription factor genes the field cancerization-specific differentially methylated regions spanned and significantly enriched GO terms.

## Data Availability

The datasets generated and/or analysed during the current study are available in the NODE repository (https://www.biosino.org/node/) (ID: OEP002659).
