# Supplementary figures and images for: Comparison of tumor and two types of paratumoral tissues highlighted epigenetic regulation of transcription during field cancerization in non-small cell lung cancer
Source: BMC Med Genomics. 2022 Mar 21;15:66. doi: 10.1186/s12920-022-01192-1 (PMC8939144; doi:10.1186/s12920-022-01192-1)

# A

## KEGG\_ECM\_RECEPTOR\_INTERACTION

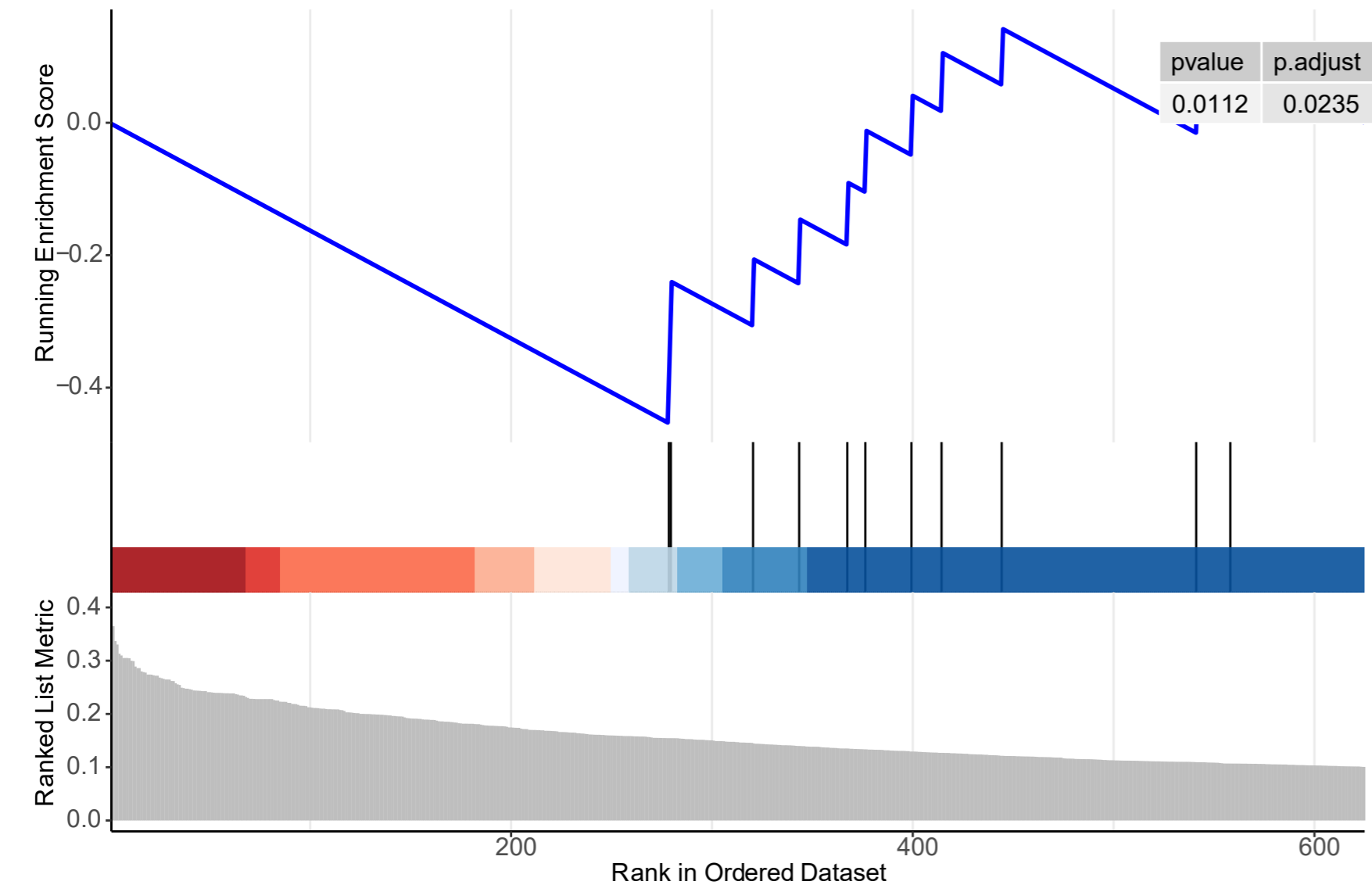

# B

## KEGG\_FOCAL\_ADHESION

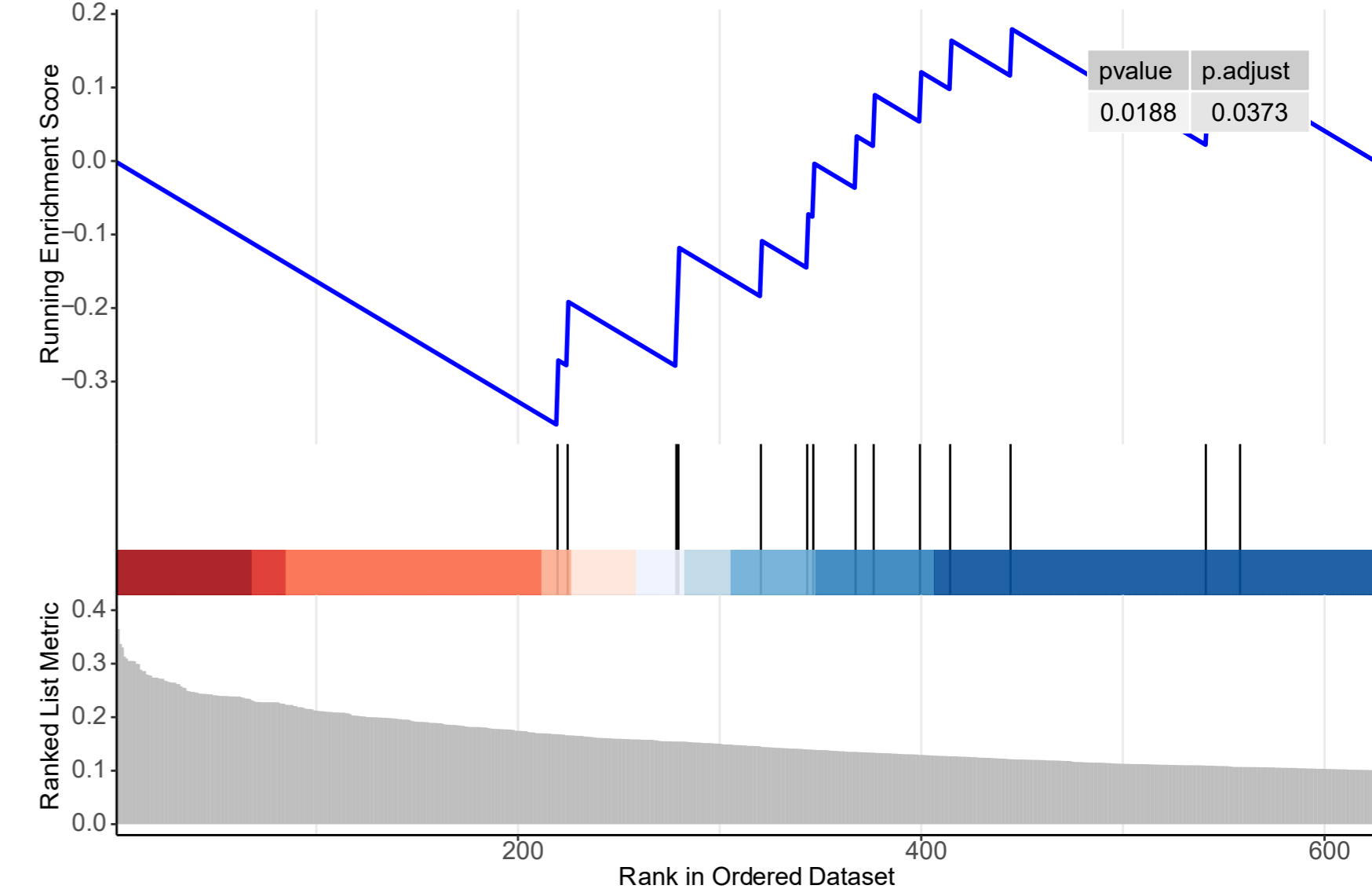

Supplement: Supplementary file 1 — Additional file 1. Fig. S1: Significantly enriched KEGG pathways terms [1] among tumor-specific differentially methylated genes using a different gene set annotation file showed similar results as in Figures 3A and 3B. All analysis parameters were the same except for the gene set file (c2.cp.v7.4.entrez.gm in this analysis). [file 12920_2022_1192_MOESM1_ESM.pdf]

# GO enrichment of hypermethylated genes (n = 626)

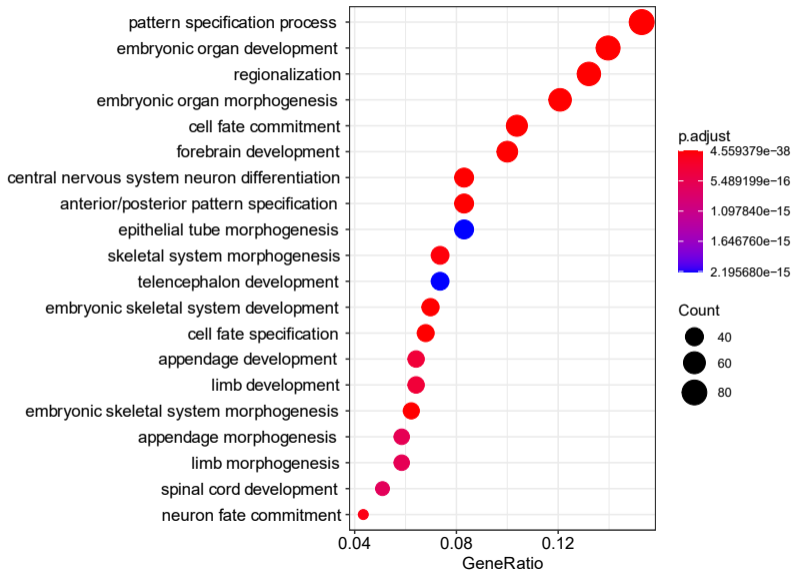

Supplement: Supplementary file 2 — Additional file 2. Fig. S2: Significantly enriched GO terms among genes harboring hypermethylated field cancerization-specific differentially methylated regions. [file 12920_2022_1192_MOESM2_ESM.pdf]

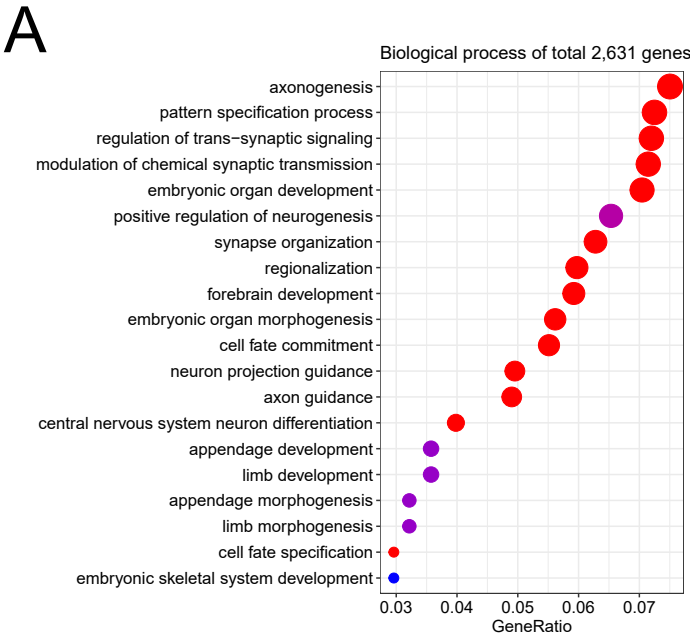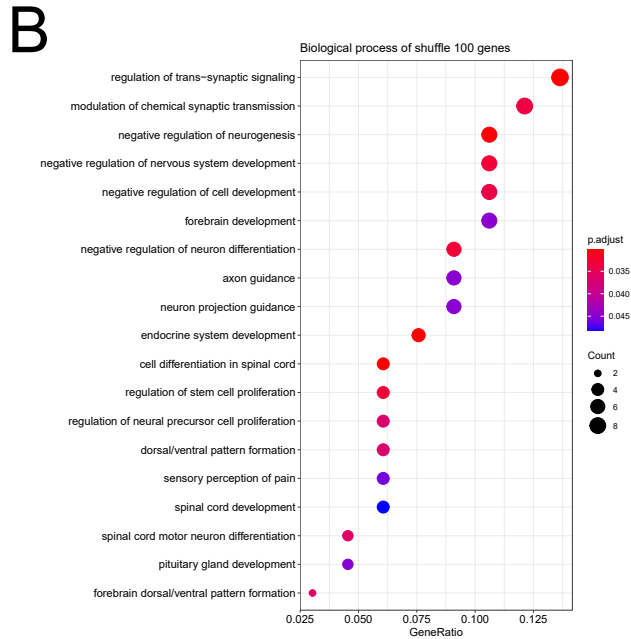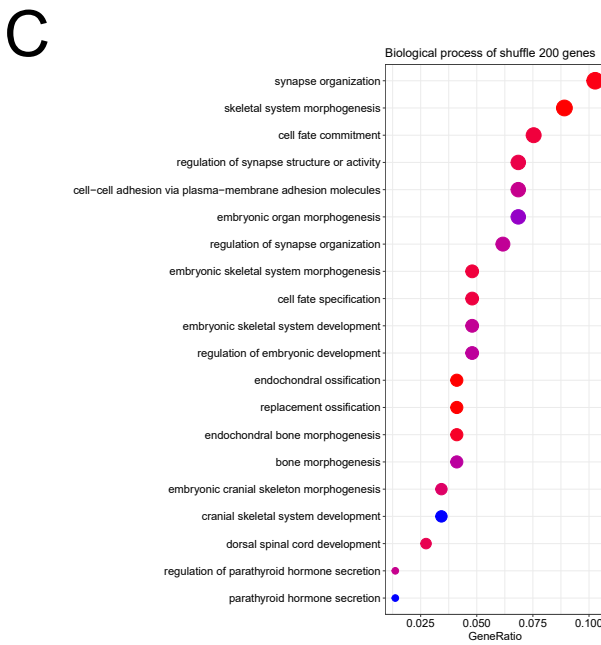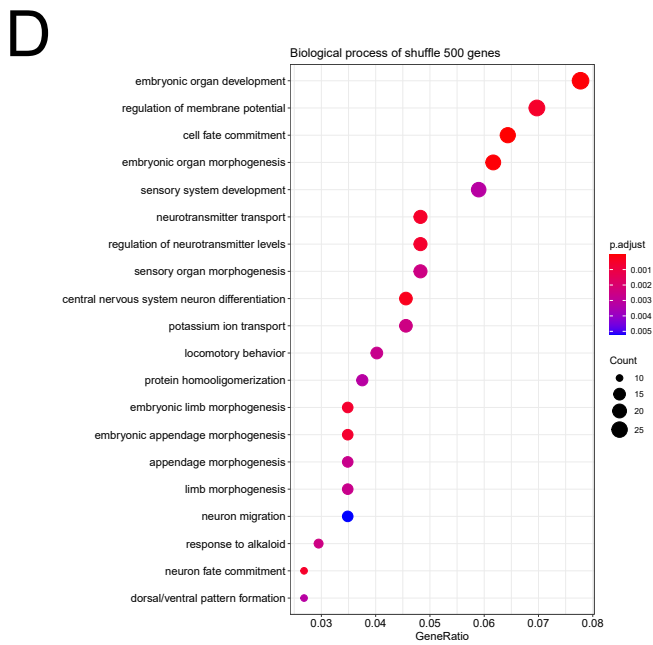

Supplement: Supplementary file 3 — Additional file 3. Fig. S3: Enriched GO biological process terms among (A) all 2613 genes that harbored the targeted methylation blocks and those among randomly sampled subsets of (B) 100, (C) 200, and (D) 500 genes. [file 12920_2022_1192_MOESM3_ESM.pdf]
